# Barriers to the Use of Trastuzumab for HER2+ Breast Cancer and the Potential Impact of Biosimilars: A Physician Survey in the United States and Emerging Markets

**DOI:** 10.3390/ph7090943

**Published:** 2014-09-17

**Authors:** Philip Lammers, Carmen Criscitiello, Giuseppe Curigliano, Ira Jacobs

**Affiliations:** 1 Meharry Medical College, 1005 Dr. D.B. Todd, Jr. Boulevard, Nashville, TN 37208-3599, USA; 2 Early Drug Development for Innovative Therapies Division, European Institute of Oncology, Via Ripamonti, 435-20141 Milano, Italy; E-Mails: carmen.criscitiello@ieo.it (C.C.); giuseppe.curigliano@ieo.it (G.C.); 3 Pfizer Inc, 235 East 42nd St, New York, NY 10017-5755, USA; E-Mail: ira.jacobs@pfizer.com

**Keywords:** trastuzumab, biosimilar, access, emerging markets, HER2+ breast cancer

## Abstract

Trastuzumab in combination with chemotherapy has become a standard of care for patients with HER2+ breast cancer. The cost of therapy, however, can limit patient access to trastuzumab in areas with limited financial resources for treatment reimbursement. This study examined access to trastuzumab and identified potential barriers to its use in the United States, Mexico, Turkey, Russia and Brazil via physician survey. The study also investigated if the availability of a biosimilar to trastuzumab would improve access to and use of HER2 monoclonal antibody therapy. Across all countries, a subset of oncologists reported barriers to the use of trastuzumab in a neoadjuvant, adjuvant or metastatic setting. Common barriers to the use of trastuzumab included issues related to insurance coverage, drug availability and cost to the patient. Overall, nearly half of oncologists reported that they would increase the use of HER2 monoclonal antibody therapy across all treatment settings if a lower cost biosimilar to trastuzumab were available. We conclude that the introduction of a biosimilar to trastuzumab may alleviate cost-related barriers to treatment and could increase patient access to HER2-directed therapy in all countries examined.

## 1. Introduction

Breast cancer is the most common cancer among women and is the primary cause of cancer death among women worldwide, accounting for ~522,000 deaths in 2012 [1]. Incidence rates differ throughout the world, but rates are highest in developed countries [1]. In the United States (US), for example, over 235,000 new cases of invasive breast cancer are expected in 2014 [2]. Survival rates also vary across regions; rates are over 80% in North America, Australia, Sweden, Finland and Japan, ~60% in Brazil, Poland and Slovakia, but below 40% in Algeria [3]. Low survival rates in less-developed countries may be explained by a lack of early detection programs and poor access to the newest therapies [4]. Even in the U.S., despite advanced detection and treatment programs, it is estimated that approximately 40,000 women will die from breast cancer in 2014 [2].

Trastuzumab, marketed under the trade name, Herceptin^®^ (Genentech, South San Francisco, CA, USA), is a monoclonal antibody directed against the human epidermal growth factor receptor 2 protein (HER2) [5]. Trastuzumab is indicated as an adjuvant treatment for HER2-overexpressing breast cancer and for the treatment of metastatic HER2-overexpressing breast cancer [5,6]. In three large-scale, randomized, open-label trials in women with HER2+ breast cancer, treatment with adjuvant trastuzumab resulted in a 52% increase in disease-free survival [7,8] and a 33% reduction in risk of death [8] relative to chemotherapy alone. Likewise, the addition of trastuzumab to standard chemotherapy in patients with first-line metastatic HER2+ breast cancer resulted in an increase in time to disease progression and resulted in a 20% decrease in the risk of death [9]. Trastuzumab, in combination with pertuzumab and docetaxel, has also shown efficacy in women with locally-advanced, inflammatory or early HER2+ breast cancer [10].

Biologics, such as trastuzumab, are complex medicines developed from living organisms [11]. The cost associated with biologic therapy may limit patient access to these medicines in areas with limited financial resources for treatment reimbursement. Decreasing the cost of therapy and, as a result, improving access to biologic treatments is a priority in such areas. The use of biosimilars may be one approach to increase access to specific biologic therapies by offering a comparable, but more affordable, alternative. The term “biosimilar” refers to a biologic drug that is developed to be highly similar, in terms of safety, purity and potency, to an existing licensed reference biologic [11,12,13]. Biosimilars treat the same conditions as the reference biologic, but are commonly priced 25% to 30% less [12,14]. The use of biosimilars is expected to grow substantially in the near future as biologic patents expire [12,13]. In anticipation of this growth and in recognition of the potential that biosimilars have to increase access to biologic therapy, the Biologics Price Competition and Innovation Act of 2009 (part of the Patient Protection and Affordable Care Act) and the European Medicines Agency Committee for Medicinal Products for Human Use have established guidelines for the development and streamlined approval of biosimilars in the U.S. [15] and the EU [16], respectively.

Access to trastuzumab may be a barrier to effective treatment for a large group of patients. With this issue in mind, the objectives of the current study were to examine the level of access that physicians and patients in the U.S., Mexico (MEX), Turkey (TUR), Russia (RUS) and Brazil (BRZ) have to Herceptin (trastuzumab) and to identify potential barriers that may prevent its use in the treatment of HER2+ breast cancer. An additional objective of this study was to determine whether the introduction of a biosimilar to trastuzumab would increase access to and use of HER2 monoclonal antibody therapy in these countries.

## 2. Experimental Section

### 2.1. Approach

A questionnaire was designed to evaluate the access to and usage of trastuzumab by oncologists in the U.S., BRZ, MEX, RUS and TUR. Recruiters who were familiar with each country’s healthcare system and culture engaged physicians. Physicians were identified from proprietary databases, association lists, telephone directories and other commercially available sources. Alternatively, physicians who were existing members of a recruiter’s online community could request to participate in the survey. Physicians were invited to participate by telephone or email. Those agreeing to participate were emailed a link to the automated online survey. A short screener survey was used to identify physicians for the main survey. To participate, physicians must have been a full-time hematologist/medical oncologist (practicing at least 30 h/week), in practice between 2 and 30 years, spent at least 75% of the time in direct patient care and have a practice consisting of a combination of hospital and office based (U.S. only), and at least 20% of their cancer patients must have been diagnosed with breast cancer (at least 10% of those patients being HER2+).

The goal was to survey 500 physicians; 200 in the U.S. and 75 each in BRZ, MEX, RUS and TUR. In total, 7,994 physicians were invited to participate in the survey (U.S. = 1,530, BRZ = 3,848, MEX = 1,529, RUS = 702, TUR = 385). The short screener survey was failed by 351 physicians, and another 16 physicians started the main survey, but discontinued without completing. Once a country achieved its pre-specified goal, the survey was no longer administered in that country, regardless of the number of invited physicians remaining/willing to participate. Using these sample sizes, the margins of error at the 95% confidence level were ±4.4% (overall), ±6.9% (U.S.; *n* = 200) and ±11.3% (all other countries; *n* = 75).

### 2.2. Survey Details

The questionnaire focused on HER2+ breast cancer patients. Specific questions were related to: breast cancer disease categories; types of patient insurance; factors considered when making treatment decisions; use of treatment guidelines; use of trastuzumab; barriers to the use of trastuzumab; and the potential use of a less expensive biosimilar version of trastuzumab if it were available. Questions were answered in a variety of forms, including: yes/no; percentage; 5- or 7-point Likert scale; and select all that apply. In some cases, participants were offered a text box to further explain their answers. The survey was expected to take ~10 min to complete, and participants received an honorarium upon completion. The survey was administered from December, 2012, to January, 2013.

## 3. Results

### 3.1. Breast Cancer Patient Population

Physicians reported that 41% (range, 36%–53%) of their patients had breast cancer, and nearly one-third (range, 25%–35%) of the cancers were characterized as HER2+ (Table 1). HER2+ breast cancer patients utilized different types of insurance coverage across markets. More than 50% of patients, particularly those in MEX, TUR and RUS, had government-funded insurance (overall = 55%, U.S. = 37%, BRZ = 34%, MEX = 69%, TUR = 93%, RUS = 74%). Twenty-two percent of patients received private medical insurance through their employer (U.S. = 38%, BRZ = 26%, MEX = 8%, TUR = 4%, RUS = 7%), while 18% (U.S. = 20%, BRZ = 36%, MEX = 16%, TUR = 2%, RUS = 8%) paid for their own private insurance. Patients in MEX and RUS were the most likely to pay for medical expenses out of pocket, compared with other markets (overall = 5%, U.S. = 5%, BRZ = 5%, MEX = 8%, TUR < 1%, RUS = 11%).

**Table 1:** Description of the breast cancer patient population.

Type of Cancer	*Overall*	U.S.	BRZ	MEX	TUR	RUS
Breast, % of all cancer patients	*41*	36	41	53	36	51
HER2+, % of breast cancer patients	*31*	35	27	28	25	33

U.S., United States; BRZ, Brazil; MEX, Mexico; TUR, Turkey; RUS, Russia.

### 3.2. Factors Considered When Treating HER2+ Breast Cancer Patients

Physicians reported that clinical treatment guidelines (88%), goals of therapy (68%) and recommendations from opinion leaders (52%) were the factors most considered when making treatment decisions regarding patients with HER2+ breast cancer. These were followed by performance status (49%), patient insurance status (39%), patient demographics (25%) and geographic locale (8%). National Comprehensive Cancer Network (NCCN) guidelines were regarded as the most influential treatment guidelines among all physicians (overall = 66%). NCCN guidelines are considered by 82% of all physicians (U.S. = 92%, BRZ = 95%, MEX = 84%, TUR = 97%, RUS = 25%) when treating patients. American Society of Clinical Oncology (overall = 58%, U.S. = 47%, BRZ = 89%, MEX = 76%, TUR = 61%, RUS = 36%), European Society for Medical Oncology (overall = 56%, U.S. = NA, BRZ = 63%, MEX = 36%, TUR = 65%, RUS = 59%) and St. Gallen (overall = 47%, U.S. = NA, BRZ = 63%, MEX = 59%, TUR = 32%, RUS = 33%) guidelines were also frequently reported as guidelines considered by physicians. National Ministry of Health guidelines were reported by 52% of physicians in RUS.

Physicians were asked whether the clinical guidelines they follow explicitly recommend the use of trastuzumab for the treatment of HER2+ breast cancer patients in different clinical settings. Across all countries, a majority of physicians reported that trastuzumab is explicitly recommended for use in a neoadjuvant setting in HER2+ breast cancer patients (overall = 57%). This was reported by a greater number of physicians in the U.S. (66%), TUR (72%) and RUS (56%) and fewer in BRZ (35%) and MEX (39%). Across all countries, a majority of physicians reported that trastuzumab is also explicitly recommended for use in an adjuvant setting in HER2+ breast cancer patients if the therapy duration is greater than 6 months (overall = 63%). This was reported by a greater number of physicians in the U.S. (69%), TUR (81%) and RUS (65%) than in BRZ (49%) and MEX (44%). Finally, a majority of physicians reported that trastuzumab is explicitly recommended for use in a metastatic setting in HER2+ breast cancer patients. This was consistent for both first line (overall = 68%, U.S. =77%, BRZ = 55%, MEX = 49%, TUR = 87%, RUS=56%) and second line (overall = 64%, U.S. = 71%, BRZ = 48%, MEX = 41%, TUR = 83%, RUS = 68%) treatment settings.

### 3.3. Trastuzumab Usage and Barriers to Treatment

Physicians were asked to report how frequently (1 = always, 2 = frequently, 3 = not so often, 4 = rarely, 5 = never) they used trastuzumab in HER2+ breast cancer patients in different clinical settings. In all countries, except RUS, a majority of physicians reported always or frequently using trastuzumab in a neoadjuvant setting (overall = 73%, U.S. = 78%, BRZ = 80%, MEX = 81%, TUR = 83%, RUS = 33%). A majority of physicians also reported always or frequently using trastuzumab in an adjuvant (overall = 92%, U.S. = 93%, BRZ = 99%, MEX = 95%, TUR = 97%, RUS = 74%) and in a metastatic (overall = 92%, U.S. = 94%, BRZ = 100%, MEX = 93%, TUR = 98%, RUS = 73%) setting. Physicians in RUS reported more infrequent use of trastuzumab in these settings than physicians from any other country.

Physicians who reported a score of 3 (not so often), 4 (rarely) or 5 (never) were asked to identify the reasons they did not use trastuzumab in different clinical settings (Table 2). Across all countries, the most common barriers to the use of trastuzumab in a neoadjuvant setting were related to insurance coverage, availability of the drug, treatment guidelines, patient comorbidities and cost to the patient. The most common barriers to the use of trastuzumab in an adjuvant setting were related to availability of the drug, cost to the patient, patient comorbidities, clinical data, insurance coverage and treatment guidelines. The most common barriers to the use of trastuzumab in a metastatic setting were related to insurance coverage, availability of the drug and treatment guidelines.

**Table 2:** Most common barriers to the use of trastuzumab (*n* (%) of physicians) ^a^^,b^.

**In a Neoadjuvant Setting**	***Overall* (*n* = 137)**	**U.S. *(n* = 45)**	**BRZ (*n* = 15)**	**MEX (*n* = 14)**	**TUR (*n* = 13)**	**RUS (*n* = 50)**
Not included in the formulary of drugs covered by patients’ insurance	*38*	33	53	50	23	62
Not available in the hospital/clinic where I practice	*37*	11	33	50	23	42
Use not recommended by treatment guidelines or protocol I follow in this setting	*37*	49	40	36	0	38
Patient has medical issues why they cannot take the drug (e.g., cardiac)	*36*	38	13	7	0	2
High out-of-pocket treatment cost for patient	*34*	31	47	21	23	16
**In an Adjuvant Setting**	***Overall*** **(*n* = 41)**	**U.S. (*n* = 14)**	**BRZ (*n* = 1)**	**MEX** ***(n* = 4)**	**TUR** ** (*n* = 2)**	**RUS** ** (*n* = 20)**
Not available in the hospital/clinic where I practice	*44*	21	0	50	0	65
High out-of-pocket treatment cost for patient	*42*	29	100	25	0	55
Patient has medical issues why they cannot take the drug (e.g., cardiac)	*39*	29	0	25	0	45
Use not supported by clinical trial data in this setting	*39*	64	0	50	100	15
Not included in the formulary of drugs covered by patients’ insurance	*37*	36	100	50	50	50
Use not recommended by treatment guidelines or protocol I follow in this setting	*32*	50	0	25	0	25
**In a Metastatic Setting**	***Overall* (*n* = 39)**	**U.S. (*n* = 12)**	**BRZ (*n* = 0)**	**MEX (*n* = 5)**	**TUR (*n* = 2)**	**RUS (*n*= 20)**
Not included in the formulary of drugs covered by patients’ insurance	*49*	42	0	20	0	40
Not available in the hospital/clinic where I practice	*44*	42	0	20	0	65
Use not recommended by treatment guidelines or protocol I follow in this setting	*41*	25	0	20	50	25

^a^ Percentages based only on those physicians who reported that they not so often, rarely or never use trastuzumab. See “*n*” in each column for the number of physicians used to calculate percentages. ^b^ Barriers reported by >10% of these physicians across all markets. U.S., United States; BRZ, Brazil; MEX, Mexico; TUR, Turkey; RUS, Russia.

Nearly one-third of all physicians, and a higher percentage in MEX, BRZ and RUS, answered yes to the question “are there any instances where you have not been able to treat a patient with trastuzumab or have had to delay their treatment due to reimbursement issues” (overall = 31%, U.S. = 10%, BRZ = 48%, MEX = 37%, TUR = 16%, RUS = 76%). The most common reasons for this inability to use trastuzumab or delay in treatment are presented in Table 3. Reimbursement issues were encountered across all clinical settings (overall: neoadjuvant = 28%, adjuvant = 39%, metastatic = 32%). The most common setting where reimbursement issues were encountered was the metastatic setting in the U.S. (60%) and the adjuvant setting in all other countries (BRZ = 39%, Mexico = 43%, TUR = 33%, RUS = 47%).

**Table 3:** Most common reasons physicians had to cancel or delay treatment with trastuzumab (% of physicians) ^a^.

Reasons for Cancel or Delay	*Overall* (*n* = 153)	U.S. (*n* = 20)	BRZ (*n* = 36)	MEX (*n* = 28)	TUR (*n* = 12)	RUS (*n* = 57)
Patient had no insurance/not eligible for reimbursement	*28*	32	22	32	39	25
Insurance/government refused to fund the treatment	*27*	32	64	11	24	10
Hospital did not have funds to provide trastuzumab	*26*	10	8	33	8	44
Patient unable to pay copayment	*15*	14	6	22	15	16
Other	*4*	12	0	2	14	5

^a^ Based only on those physicians who reported having to cancel or delay treatment with trastuzumab. U.S., United States; BRZ, Brazil; MEX, Mexico; TUR, Turkey; RUS, Russia.

### 3.4. Impact of a Biosimilar to Trastuzumab

Across all markets, 45% of physicians reported that they would increase the use of HER2 antibody therapy if a lower cost biosimilar version of trastuzumab were available (U.S. = 29%, BRZ = 53%, MEX = 63%, TUR = 23%, RUS = 81%). This trend was observed across all clinical settings (Table 4). Among physicians who said a biosimilar version would not increase their use of HER2 antibody therapy, the most common reason was reported as “already always use it in all the appropriate patients/situations” (overall = 35%).

**Table 4:** Percentage of physicians who would increase their use of HER2 antibody therapy in different clinical settings if a biosimilar to trastuzumab were available ^a^.

	*Overall* (*n* = 223)	U.S. (*n* = 58)	BRZ (*n* = 40)	MEX (*n* = 47)	TUR (*n* = 17)	RUS (*n* = 61)
**Neoadjuvant setting**	*46*	52	33	53	71	38
**Adjuvant setting**						
**Duration >6 months**	*45*	45	53	43	59	38
**Metastatic setting**						
**1st line**	*50*	53	43	53	53	48
**2nd line**	*50*	59	38	32	71	57

^a^ Based only on those physicians who reported potential increased use of HER2 antibody therapy if a biosimilar were available. U.S., United States; BRZ, Brazil; MEX, Mexico; TUR, Turkey; RUS, Russia.

## 4. Discussion

This study demonstrates that there are barriers to the use of trastuzumab across the globe due to several physician-identified factors, including access to and cost of treatment. However, almost half of physicians surveyed indicated that the use of HER2-directed therapies would increase with the availability of a lower cost biosimilar HER2 monoclonal antibody.

The healthcare systems for the countries included in this study are varied, though they each offer both government-subsidized and private coverage options for their citizens. The Turkish system is predominantly government-based and managed by the Ministry of Health, though reforms over the past decade have resulted in modestly increased access to private healthcare coverage [17]. Centralized coverage is funded by a social security system that is based on employee, employer and government contributions [17]. In addition, low income citizens receive free basic coverage under the Green Card Scheme [17]. RUS also has a predominantly state-run system, managed by the Ministry of Health and Social Development, which mandates nominally free basic healthcare for all citizens [18]. Funding for this system results from a relatively even mix of tax revenues, payroll contributions and out-of-pocket payments for uncovered services (such as outpatient prescription medicines) [18]. BRZ mandates universal free healthcare for its citizens that is funded at the federal, state and local levels [19]. However, as a result, quality of care varies greatly in different regions of BRZ, and approximately 25% of Brazilians opt to purchase private medical insurance [19]. The Mexican healthcare system is also a mix of government-subsidized (amount of subsidy based on patient income level) and private coverage [20]. The U.S. healthcare system is based largely on private insurance coverage, purchased by the patient, though government-subsidized options are available to older citizens, citizens with certain disabilities and citizens with low income. Recent healthcare reform in the U.S. requires most citizens to purchase some form of basic healthcare coverage [21].

Despite different healthcare systems, barriers to the use of trastuzumab were identified in every country examined. Though the study was not powered to directly compare different countries, certain assumptions can be made based on the data presented. BRZ, for example, had the least number of patients with government-funded healthcare and the greatest number of patients having to pay for private medical insurance on their own. Likely because of this, physicians from BRZ most often cited “high out-of-pocket costs for the patient” as a barrier to the use of trastuzumab. Though ~70% of patients in MEX and RUS had government-funded healthcare, physicians in these countries most often reported that trastuzumab was “not available in the hospital/clinic where they practice” or “not included in the formulary of drugs covered by patient’s insurance” as barriers to the use of trastuzumab. Not surprisingly, physicians from BRZ, MEX and RUS most often reported having to cancel or delay treatment with trastuzumab due to reimbursement/payment issues. Even in the U.S., where private medical insurance is predominate, physicians commonly reported “high out-of-pocket costs for the patient”, trastuzumab “not being included in the formulary of drugs covered by patient’s insurance” or trastuzumab “not being available in the hospital/clinic where they practice” as barriers to the use of trastuzumab. Overall, these data demonstrate that the cost of treatment is a significant barrier to the access and use of trastuzumab. In addition, clinical treatment guidelines were often cited as a barrier to the use of trastuzumab, particularly in an adjuvant or metastatic treatment setting.

The costs of cancer care are increasingly becoming important to the U.S. payer system, as cost containment is a key component of the Affordable Care Act [15]. A study comparing the treatment cost of biosimilars *vs.* innovator biologic for five common treatments in India estimated that the use of biosimilars would result in yearly savings of ~843 million U.S. dollars [22]. Reditux™ (Dr. Reddy’s Laboratories, Hyderabad, India), for example, the first intended biosimilar to the monoclonal anti-CD20 cancer agent rituximab, was launched in India in 2007 at 50% lower cost than the originator biologic (MabThera™^®^, Hoffman-LaRoche, Basel, Switzerland). As a result, patient access to CD-20-directed therapy increased six-fold within three years of launch [23]. Likewise, the availability of a trastuzumab biosimilar may alleviate the cost-related barriers to the access and use of HER2 antibody therapy. Many oncologists clearly understand the cost benefit of biosimilars, as a majority of those surveyed from the U.S. and the EU expect to prescribe biosimilars to common monoclonal antibody treatments within the first 12 months of their launch [24].

In addition to HER2+ breast cancer patients, other patient populations may also benefit from the availability of a biosimilar to trastuzumab. Trastuzumab is also indicated for the treatment of HER2-overexpressing metastatic gastric or gastroesophageal junction adenocarcinoma, and its addition to chemotherapy significantly improves overall survival [25]. In the future, these patients may also benefit from the availability of a biosimilar to trastuzumab. However, the current study does not address the use of trastuzumab or the availability of a biosimilar in these malignancies.

All survey research, including the current study, is limited in some way. For example, respondents (physicians in this case) may not precisely recall experiences in the past. Additionally, the survey question regarding the use of a potential biosimilar to trastuzumab is limited in that it does not specify exactly how much less expensive the biosimilar would be compared to Herceptin^®^. In order to achieve our survey responder goal of 500 physicians (200 U.S. and 75 each in MEX, BRX, RUS and TUR), nearly 8,000 total physicians were invited to participate. It is possible that our findings are subject to non-responder bias, since a majority of invited physicians did not participate or were unable to participate due to closure of the survey once the responder goal was reached. Though the physicians and countries included in this survey vary in their geographic, economic, cultural and healthcare environment, care must be taken when attempting to generalize our findings to physicians from countries outside the scope of this study.

## 5. Conclusions

Innovations in cancer therapy are costly and contribute to rising healthcare expenditures. Improved accesses to therapies and novel delivery models have the potential to advance the prevention and treatment of cancer. The introduction of high-quality biosimilars could potentially benefit patients by broadening access to alternative biologic therapies. In this study, we conclude that there are significant barriers to the use of trastuzumab and that a biosimilar to trastuzumab could increase access to and use of HER2-directed antibody therapy in patients with HER2+ breast cancer.
